# From Congenital Disorders of Fat Malabsorption to Understanding Intra-Enterocyte Mechanisms Behind Chylomicron Assembly and Secretion

**DOI:** 10.3389/fphys.2021.629222

**Published:** 2021-01-27

**Authors:** Emile Levy, Jean François Beaulieu, Schohraya Spahis

**Affiliations:** ^1^Research Centre, CHU Ste-Justine, Université de Montréal, Montreal, QC, Canada; ^2^Department of Nutrition, Université de Montréal, Montreal, QC, Canada; ^3^Department of Pediatrics, Université de Montréal, Montreal, QC, Canada; ^4^Laboratory of Intestinal Physiopathology, Faculty of Medicine and Health Sciences, Université de Sherbrooke, Sherbrooke, QC, Canada

**Keywords:** fat digestion, lipid absorption, congenital malabsorption syndromes, chylomicron, intestine

## Abstract

During the last two decades, a large body of information on the events responsible for intestinal fat digestion and absorption has been accumulated. In particular, many groups have extensively focused on the absorptive phase in order to highlight the critical “players” and the main mechanisms orchestrating the assembly and secretion of chylomicrons (CM) as essential vehicles of alimentary lipids. The major aim of this article is to review understanding derived from basic science and clinical conditions associated with impaired packaging and export of CM. We have particularly insisted on inborn metabolic pathways in humans as well as on genetically modified animal models (recapitulating pathological features). The ultimate goal of this approach is that “experiments of nature” and *in vivo* model strategy collectively allow gaining novel mechanistic insight and filling the gap between the underlying genetic defect and the apparent clinical phenotype. Thus, uncovering the cause of disease contributes not only to understanding normal physiologic pathway, but also to capturing disorder onset, progression, treatment and prognosis.

## Introduction

Intestinal fat transport is a prerequisite process to deliver alimentary lipids to the bloodstream for subsequent metabolism and peripheral energy homeostasis. Several biochemical, physiological and morphological requirements must be met to allow intraluminal digestion and intracellular transport of triglycerides (TG), phospholipids (PL), and cholesterol (CHOL). Digestive processing and mucosal transport represent the essential steps to warrant optimal lipid absorption. However, their abnormalities result in intestinal fat malabsorption not only of lipids, but also of fat-soluble vitamins, concomitantly with gastrointestinal (GI) symptoms along with steatorrhea, nutritional fatty acid (FA) and vitamin deficiency, and substantial extra-intestinal disorders. The objective of the present review is to focus on congenital disorders of intestinal lipid absorption, highlighting the molecular genetics and pathophysiological mechanisms while describing the clinical manifestations and management of patients.

## Brief Summary of Intestinal Lipid Digestion

Lipid digestion involves breakdown of TGs into FA and 2-monoglyceride (MG) by lipase, hydrolysis of cholesteryl ester (CE) into free CHOL and FA by CHOL esterase, and decomposition of PL into lysoPL and FA by phospholipase A2 in the intestinal lumen (Levy et al., 2007). The three enzymes are synthesized by the pancreas and are delivered through its exocrine acinar cells. Importantly, an alkaline mixture composed of water and bicarbonate is released by pancreatic ductal cells into duodenum to maintain the ideal pH for enzyme activity. Finally, bile salts that are concentrated in gallbladder stores also flow into the duodenum. Their detergent action assists in breaking down large fat globules into small droplets, and in solubilizing the lipids by forming micelles, thereby allowing the enzymes to get to emulsified lipid droplets. Impairment of enzymatic activity, bicarbonate supply or bile acid output leads to malabsorption symptoms, including steatorrhea, deficiency of essential FAs (EFA) and fat-soluble vitamins (A, D, E, and K), weight loss, abdominal discomfort, and abdominal bloating. Various causes and conditions may be implicated such as chronic pancreatitis, pancreatic duct obstruction, pancreatic cancer, diabetes mellitus, partial or total pancreatectomy, cystic fibrosis, inflammatory bowel diseases, small bowel resections, or bariatric intervention.

## Overview of Lipid Absorption and Chylomicron Formation

**FIGURE 1:**
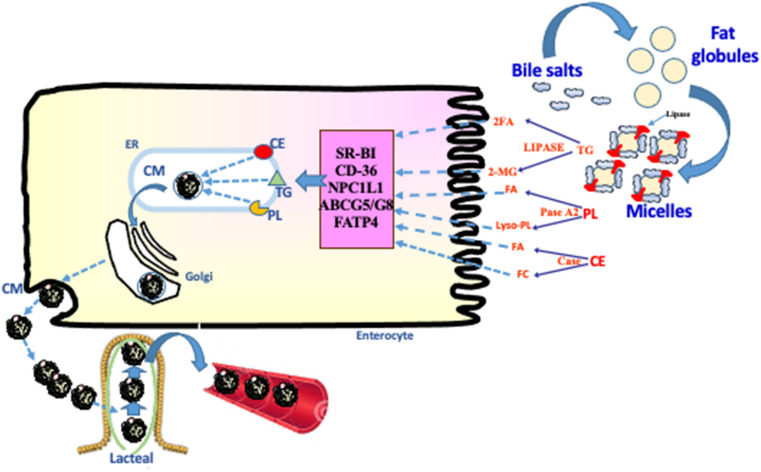
Dietary lipid digestion and absorption. Digestion of dietary fat requires bile acids, digestive enzymes and bicarbonate. Dietary lipids, mostly triacylglycerols (TG) but also cholesteryl ester (CE) and phospholipids (PL), are initially emulsified by bile acids and then hydrolyzed by pancreatic lipase, cholesterol esterase and phospholipase A2. The lipolytic products fatty acids (FA), 2-monoglyceride (2-MG), free cholesterol (FC), and lyso PL form micelles by the action of bile acids in the proximal small bowel. The micelles release the lipolytic products near to the microvillus membrane to allow their uptake by lipid transporters: scavenger receptor BI (SR-BI), fatty acid translocase (CD-36), Niemann-Pick C1-Like 1 (NPC1L1), ATP-binding cassette G5/G8 (ABCG5/G8), and fatty acid transport protein 4 (FATP4). Following their uptake, the lipolytic products are deposited in the endoplasmic reticulum (ER) by L- and I- fatty acid binding proteins (L- and I-FABP), for esterification, assembly with apolipoproteins, packaging into chylomicrons (CM) and transport to bloodstream via the lymphatics.

Following intraluminal fat digestion, the lipolytic products must cross small intestinal brush border to form lipid-carrying lipoproteins (Figure 1). The access of FAs to enterocytes can be achieved by passive diffusion displaying a “flip-flop” pattern down a favorable concentration gradient (Mashek and Coleman, 2006; Storch and Thumser, 2010). A second FA uptake process involves protein-facilitated FA transfer in view of its saturation nature as exemplified by FA transfer proteins (Storch and Thumser, 2010). Apical microvillus membrane proteins such as fatty acid transport protein 4 (FATP4) and CD36 mediate the FA transfer inside the enterocyte (Stahl et al., 1999; Nassir et al., 2007). FATP4 is highly expressed in villus enterocytes, functions in FA incorporation, and traps FA through their conversion into CoA derivative given its endogenous acyl CoA synthetase activity (Milger et al., 2006). For its part, CD36 is also abundant in the small intestine, facilitates FA uptake, and exhibit various functions in lipid absorption such as fat test perception and food intake (Laugerette et al., 2005; Martin et al., 2011; Pepino et al., 2012).

**FIGURE 2:**
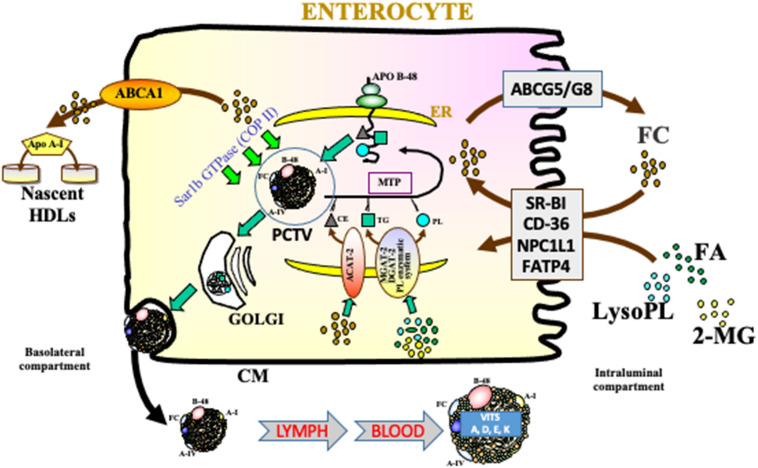
Intracellular network required for lipid transport and lipoprotein assembly. Free cholesterol (FC), monoacylglycerols (MG), lysophospholipids (LysoPL), and fatty acids (FA), carried by bile salt mixed micelles in the intestinal lumen, cross the unstirred water layer and are captured by specific proteins within brush border membranes of the jejunum considered as the optimal segment for lipid absorption. Whereas NPC1L1, viewed as the most putative transporter, mediated the uptake of digestive FC, physiologically excessive amounts are secreted back to the intestinal lumen via the apical membrane of the enterocyte by the ABCG5/G8 heterodimer. Similarly, ABCA1, localized on the basolateral surface contributes to cholesterol homeostasis by promoting cholesterol efflux to plasma apo A-I, which enhances the formation of nascent HDL. Following the transfer of lipolytic products to the endoplasmic reticulum (ER), local acyl-CoA:cholesterol *O*-acyltransferase 2 (ACAT-2) catalyzes the esterification of FC, while monoacylglycerol acyltransferase (MGAT)-diacylglycerol acyltransferase (DGAT) complex recycles FA and MG into triacylglycerols (TG), and phosphoglyceide-synthesizing enzymes intervene in phospholipids (PL) formation. Under the action of microsomal triglyceride transfer protein (MTP), lipids are then assembled with newly synthesized apo B-48 to generate chylomicrons (CM). These lipoprotein particles are then conveyed in specialized vesicles from the ER to the Golgi apparatus with the crucial participation of Sar1b GTPase. They are finally exocytosed after the fusion of Golgi vesicles and basolateral membrane.

For the intestinal transport of alimentary CHOL, various microvillus transporters have been described, including Niemann-Pick C1-Like 1 (NPC1L1), scavenger receptor-BI (SR-BI), and CD36. While these transporters regulate CHOL influx, the heteromeric complex ATP-binding cassette G5 (ABCG5)/G8 on villus brush-border membrane is in charge of CHOL efflux directly toward intestinal lumen. Besides, ABCA1 is a basolateral efflux pump that transfers CHOL to apolipoprotein (Apo) A-I, thereby contributing to high-density lipoprotein (HDL) particles (Figure 2).

Through the action of these transporters, lipolytic products move into the enterocyte. With the assistance of cytosolic binding proteins such as intestinal fatty acid-binding protein (I-FABP) and Liver-FABP (L-FABP), they are directed to the membrane of the endoplasmic reticulum (ER), where they are re-esterified. The 2-MG and FAs are reconstituted to form TG by the MG pathway involving monoacylglycerol transferase (MGAT) and diacylglycerol transferase (DGAT) (Bell and Coleman, 1980). Sequentially, MGAT catalyzes the formation of diglyceride (DG) whereas DGAT catalyzes the final reaction leading to TG. Diglycerides can also be synthesized by a secondary glycerol phosphate pathway (Lehner and Kuksis, 1996). For their part, CHOL and PL are re-esterified in their original forms by acylcholesterol acyltransferase-2 (ACAT2) and biosynthetic pathways (e.g., lyso-phosphatidylcholine acyltransferases), respectively (Buhman et al., 2000; Li et al., 2015). Thereafter, microsomal triglyceride transfer protein (MTTP) shuttles TG, CE, and PL to the structural Apo B-48 to promote chylomicron (CM) assembly (Black, 2007; Levy et al., 2011). While MTTP appears as an essential protein to uphold CM biogenesis by lipidation of the critical Apo B-48, Sar1B GTPase is another crucial component of COPII vesicles that buds from the ER to transport pre-CM to the Golgi apparatus (Levy et al., 2011). In this intracellular compartment, CM particles fuse into another transport vesicle and are vectorially transported to the basolateral membrane for secretion into the circulation via the lymphatic system (Lo and Coschigano, 2020) (Figure 1).

## Genetic Defects in Intra-Enterocyte Lipid Trafficking and Chylomicron Formation

In this section, we will review how genetic abnormalities may hamper intracellular lipid movement and CM assembly/output.

### Abetalipoproteinemia

Abetalipoproteinemia (ABL) is a homozygous autosomal recessive disorder caused by mutations of the *MTTP* gene (Wetterau et al., 1992; Shoulders et al., 1993) (Table 1). The MTTP is in fact a soluble microsomal heterodimer consisting of a unique large 97 kDa protein conferring lipid transfer activity, and the multifunctional 58 kDa protein disulfide isomerase necessary to maintain the catalytically active structure (Wetterau and Zilversmit, 1984). MTTP resides in the ER lumen and orchestrates the transfer of TG, CE, and PL onto Apo B-48 to produce pre-CM particles. Very often, Apo B-48 is not detected in the intestinal tissue of ABL patients probably due to the limiting availability of lipids, which normally protect it from proteasome degradation (Boren et al., 1992; McLeod et al., 1994; Rustaeus et al., 1995). In fact, the addition of a core lipid to the nascent Apo B-48 in the ER takes place during its translation and translocation, which prevents its degradation and allows the protein to grow and translocate completely into the lumen (Boren et al., 1994; Swift, 1995).

**TABLE 1:** Genetic defects of genes associated with intestinal malabsorption and lipid dysmetabolism along with related complications.

**Gene**	**Disease**	**Inheritance**	**Prevalence**	**Phenotype**
*MTTP*	Abetalipoproteinemia	AR	<1/1,000,000	5th Apo B and LDL Growth delay, Fat malabsorption, Hepatomegaly, Neurological dis., Neuromuscular dis.
*APO B*	Hypobetalipoproteinemia	ACD	<1/1,000,000	Φ Apo B and LDL Growth delay, Fat malabsorption, Hepatomegaly, Neurological dis., Neuromuscular dis.
Sar1B	CM retention disease	AR	<1/1,000,000	Φ CM secretion Low Apo B and LDL, Growth delay, Fat malabsorption, Hepatomegaly, Neurological dis., Ophthalmologic dis., Malnutrition
DGAT1	Congenital diarrhea Type 7	AR	ND	Malabsorption of nutrients/electrolytes, Diarrhea, FTT, hypoalbuminemia, Hypertriglyceridemia, PLE, anemia, vitamins (D and E) deficiencies
DGAT2	Charcot-Marie-Tooth disease	ACD	ND	Hypotriglyceridemia, Hypotriglyceridemia, Distal muscle weakness of the lower limbs, Sensory ataxia, Romberg sign, Decreased reflexes deep tendons
PCSK9	LOF	ACD	Prevalence* 10,25%	Low Apo B & LDL, Reduced CVD risk
ANGPTL3	Familial combined	AR	1/382,000	Hypolipidemia
NPC1L1	LOF	ND	ND	Low cholesterol, Low LDL

Most of these disorders are very rare. ACD, autosomal codominant; AR, Autosomal recessive; Dis, disorder; FTT, failure-to-thrive; LOF, loss of function; ND, no data; PLE, protein-losing enteropathy. *Noto et al. (2012).

Apart from the MTTP residence in the ER, the occurrence of membrane-associated Apo B in the Golgi, coupled with its interaction with active MTTP, suggests an important role for the Golgi in the biogenesis of Apo B-containing lipoproteins (Levy et al., 2002). As the liver proceeds in the same way to assemble very low-density lipoprotein (VLDL), the genetic mutations of *MTTP* affects Apo B-100-containing hepatic derived lipoproteins. It is important to remember that the two Apo B translation products (B-100 and B-48) are produced by the same gene, but intestinal Apo B-48 is generated through mRNA editing mechanism employing the catalytic deaminase APOBEC1 (Teng et al., 1990, 1993; Anant et al., 1995).

In view of the aberrations of *MTTP*, there is a total absence of circulating CM (following fat feeding) and Apo B-containing lipoproteins along with extremely low level of plasma TG, total CHOL, and Apo B-100 (Raabe et al., 1998). Young children present with diarrhea, malabsorption, and severe steatorrhea, with additional features such as deficiency of EFAs and fat-soluble vitamins, red blood cell acanthocytosis, retinal degeneration, and neurological dysfunction (likely due to the paucity of vitamins A, D, E, K), and steatohepatitis (Black et al., 1991).

Given the failure to thrive and abnormal clinical features such as neurological sequelae in early childhood, genetic testing should rapidly validate the diagnosis. ABL patients necessitate permanent maintenance on a low-fat diet. Although long-term high-dose supplementation with vitamins A (10–15,000 IU/day) and E (100 mg/kg/day) improve retinal and neurological functions (Granot and Kohen, 2004), their plasma levels seldom return to the normal range (Traber, 2013).

### Hypobetalipoproteinemia

Familial hypobetalipoproteinemia (FHBL) is a monogenic, inherited disorder, which closely resembles ABL, but is essentially caused by genetic defects of the *APOB* gene on chromosome 2 (Whitfield et al., 2003) (Table 1). As a result of non-sense, frame shift and splicing mutations in the *APOB* gene, prematurely truncated Apo B forms (i.e., smaller proteins than Apo B) are associated with the total absence of circulating CMs and Apo B-48 in response to fat meals (Levy et al., 1994). Moreover, the liver is unable to secrete VLDL, and extremely low plasma concentrations of low-density lipoprotein (LDL), TG and total CHOL are common in FHBL (Di Leo et al., 2008; Buonuomo et al., 2009). Sometimes, search of mutations uncovered truncated *APOB* forms of various lengths, ranging from *APOB-6.46* to *APOB-89*. The truncated forms are characterized by the missing carboxyl-terminal portion, which must have interfered with the translation of full-length *APOB* (Wang et al., 2018).

Homozygous FHBL presents in infancy or early childhood with variable clinical manifestations, including failure to thrive, steatorrhea, undetectable fat-soluble vitamins, EFA deficiency, acanthocytosis, and neurologic deficits with macular degeneration (Lee and Hegele, 2014). In adulthood, FHBL may be affected by hepatic steatosis (Heeks et al., 2013). As can be seen, the phenotype is similar to that of ABL, and the same goes for management. Unfortunately, homozygous FHBL may be accompanied by cirrhosis (Bonnefont-Rousselot et al., 2009; Florkowski et al., 2010) and hepatocellular carcinoma (Di Leo et al., 2008; Cefalu et al., 2013). It is therefore recommended that patients undergo hepatic evaluation regularly.

### Chylomicron Retention Disease (CRD)

CRD is another congenital malabsorption disorder that highlights the obligatory trafficking of nascent CM between ER and Golgi in intestinal absorptive cells (Levy et al., 1987; Roy et al., 1987) (Table 1). Mutations of *SAR1-ADP ribosylation factor, type B* (*SAR1B*) prevent the conveyance of CM-containing vesicles through the early secretory pathway, leading to the accumulation of pre-CM (Jones et al., 2003; Charcosset et al., 2008). In fact, *SAR1B*, belonging to the Ras superfamily of guanosine triphosphatases (GTPases), is essential for the coatomer COPII that transports proteins from the rough ER to the Golgi apparatus, a process requiring the small Sar1b GTPase for the exchange of GDP for GTP. Important studies have shown the ability of *SAR1B* to initiate vesicle formation by recruiting first the inner COPII coat components (Sec23 and Sec24) and subsequently the components of the outer flexible coat (Sec13/Sec31) (Barlowe et al., 1994). It has been proposed that CMs of large size move from the ER to Golgi, probably inside the pre-CM transport vesicle (PCTV) (Siddiqi et al., 2003, 2006). From where we stand at present, *SAR1B* aberrations affect the transport of pre-CM from the ER to the Golgi in PCTV, including their fusion with the cis-Golgi. Nevertheless, further studies are explicitly required to improve our vision of the mechanisms implicated in CRD pathogenesis.

Chronic diarrhea, vomiting, abdominal distension, and failure to thrive are among the most frequent and earliest symptoms affecting CRD patients. Incapacity to export CM in CRD impairs the intestinal transport of fat-soluble vitamins and the status of EFA. Furthermore, plasma levels of CHOL, PL, LDL, HDL, and Apos (B, A-I) are usually below 50% of control values (Peretti et al., 2009, 2010). Additional clinical findings comprise ophthalmologic (micronystagmus, mild deficit in the perception of the blue yellow axis and delayed dark adaptation) and neurological complications (areflexia, proprioceptive aberrancy, ataxia, myopathy, and sensory neuropathy), which are of lesser importance compared with those of ABL and FHPL. Elevated creatine kinase and cardiomyopathy have also been reported along with muscular abnormality. Also noteworthy are the inadequate mineralization and retarded bone maturation. While moderate hepatomegaly and macrovesicular steatosis are detected, steatohepatitis, and cirrhosis remain rare (Peretti et al., 2010).

Although clinical examination and biological evaluations centring on nutrition growth, GI, liver and neurological manifestations may help in the diagnosis, genetic testing (identifying Sar1B mutations) remains the most accurate and reliable tool. Importantly, the signs of chronic diarrhea, fat malabsorption, fat-laden enterocytes, atypical lipid, and vitamin profile are suggestive of CRD.

Management of these patients consists in recommending a fat-free diet, enriched in EFA, medium-chain TGs and liposoluble vitamins, including 50 UI/kg/day vitamin E, 15,000 IU/day vitamin A, 15 mg/week vitamin K, and 800–1,200 UI/kg/day or 100,000 IU/2 months vitamin D if younger than 5 years old and 600,000 IU/2 months if older than 5 years old (Peretti et al., 2010). Recently, a study evaluated the efficacy of fat-soluble vitamin E acetate and tocofersolan (a water-soluble derivative of RRR-α-tocopherol) by evaluating the ability of each formulation to restore vitamin E storage after 4 months of treatment (Cuerq et al., 2018). While in patients with ABL, tocofersolan and α-tocopherol acetate bioavailability was extremely low (2.8 and 3.1%, respectively), bioavailability was higher in patients with CRD (tocofersolan, 24.7%; α-tocopherol acetate, 11.4%).

### CD36 Alteration and Intestinal Lipid Secretion

FA translocase or CD36 is a class B scavenger receptor, which is anchored in the membrane by transmembrane domains, and is largely involved in high affinity FA uptake in several tissues (Yamashita et al., 2007). In the small intestine, CD36 is localized in the villus membrane of the jejunum (Poirier et al., 1996; Chen et al., 2001; Lobo et al., 2001). Knockout (KO) of CD36 did not disturb the intestinal uptake of FA, and no impaired FA absorption was seen in CD36-KO mice (Goudriaan et al., 2002). Later, it was discovered a reduced lipid output in the lymph of CD36-null mice (Drover et al., 2005). Probably, the reason for this inconsistency was that CD36 ablation also impacts on CM clearance by affecting its size (Drover et al., 2005), resulting in circulating CM build-up, thereby concealing the lessened lipid output from the enterocytes to the lymphatic system. The presence of CD36 is therefore necessary for the delivery of lipids from the gut. Accordingly, patients with CD36 deficiency exhibited increased levels of plasma TG, free FA, CM remnants, and Apo B-48 because of an enhanced production of smaller lipoproteins than CM in the intestine (Masuda et al., 2009).

### FATP4 Alteration and Intestinal Lipid Secretion

To elucidate the role of FATP4 in intestinal FA uptake, *Fatp4* knockdown is performed in primary mouse small intestinal enterocytes. *FATP4* deficiency reduces FA uptake (Stahl et al., 1999). Seemingly, this downregulation is not dependent on the transport function of the FATP4 protein but rather on its enzymatic activity conducting to FA esterification with coenzyme A for TG and phospholipid biosynthesis in the gut (Milger et al., 2006; Ko et al., 2020). If heterozygous *Fatp4*^+/–^ mice displays reduced long-chain FA uptake (Gimeno et al., 2003), Fatp4^–/–^ knockdown mice mates display similar food intake, growth, weight gain, intestinal TG absorption and fecal fat loss on either low or high-fat diets (Shim et al., 2009). Although serum CHOL concentrations were lower in *Fatp4*^–/–^ mice, the authors conclude that intestinal FATP4 has no physiological part in dietary lipid absorption in mice. However, in the presence of bacterial infection causing intestinal nutrient malabsorption in piglets, a synthetic antimicrobial peptide KR-32 alleviates malabsorption by improving the expression of FABP4 (Liu et al., 2019).

As G/A polymorphism in exon 3 of the *FATP4* genes rise to a Gly209Ser substitution with potential structural-functional implications, a group of researchers investigates whether variation within the FATP4 gene influences fasting and postprandial lipid and lipoprotein variables along with markers of insulin resistance (IR) in healthy, middle-aged Swedish men (Gertow et al., 2004). Their hypothesis turns out to be correct in view of the negative association of the *FATP4* variant with metabolic syndrome components, including IR, TGs, postprandial lipemia, and HDL-CHOL.

### I-FABP (FABP2) Alteration and Intestinal Lipid Secretion

The intestinal form, I-FABP, is encoded by the *FABP2* gene, and is expressed exclusively in the proximal intestine where the bulk of fat absorption occurs (Thumser and Storch, 2000; Levy et al., 2009). Apparently, this protein is involved in intracellular targeting of FA given its involvement in FA transfer between membranes to allow FA metabolism and processing of dietary long-chain FA into CMs (Thumser and Storch, 2000; Hussain et al., 2005; Montoudis et al., 2008), while being regulated by lipids, hormones, and cytokines (Dube et al., 2001; Carrier et al., 2004). Accordingly, a reduction of body weight was noticed in *I-FABP*^–/–^ mice (Gajda et al., 2013), suggesting possible fat malabsorption. When *I-FABP* KO mice were challenged with high-fat diet, total fecal excretion per gram of food intake was increased concomitantly with decreased energy absorption (Lackey et al., 2020). It finally appeared that intestinal transit and motility are stimulated by *I-FABP* deletion as a consequence of altered vagal tone induced by reduced cannabinoid receptor 1 activation, thereby affecting nutrient and lipid absorption (Lackey et al., 2020). Despite all these interesting data, I-FABP overexpression in normal molecularly modified normal human intestinal epithelial cells is not related to lipid esterification, Apo synthesis and lipoprotein assembly, which therefore excludes its role in intestinal fat transport (Montoudis et al., 2006). Likewise, although I-FABP shows a high affinity for long-chain FAs and has been suggested to be involved in enterocyte FA uptake (Murphy et al., 1996; Alpers et al., 2000), animal models lacking *I-FABP* did not exhibit impaired FA uptake (Vassileva et al., 2000; Lagakos et al., 2011; Gajda et al., 2013). On the other hand, decreased amounts of I-FABP are observed in patients with ABL and CRD in link with the pathological intracellular accumulation of lipid structures in the enterocytes, leading the investigators to hypothesize that I-FABP acts as a lipid sensor to prevent the intracellular esterification of FA into TGs which would otherwise lead to further additional intestinal injury (Guilmeau et al., 2007).

Importantly, some studies have shown that variations in *FABP2* gene may influence both intestinal lipid absorption and metabolism. The G-to-A substitution at codon 54 of the *FABP2* gene, which results in an alanine-to-threonine substitution at amino acid 54 (Ala54Thr) of I-FABP, has been reported to be associated with increased intestinal fat absorption (Agren et al., 1998; Levy et al., 2001), as well as FA oxidation, IR, and diabetes (Baier et al., 1995). Furthermore, it has been proposed that the effects of *FABP2* allelic variations on lipid traits are context dependent, indicating that this variant may play an important role in cardiovascular pathogenesis in the presence of IR and dyslipidemia (Stan et al., 2005). In the light of all these observations, I-FABP is central in intestinal physiology and metabolic disorders, but additional efforts are needed to precise its specific functions.

### L-FABP Alteration and Intestinal Lipid Secretion

L-FABP is abundantly detected in the small intestine and has broad FA binding specificity with high affinity for long-chain polyunsaturated FAs though it may bind to CHOL, acyl-CoA, bile acid, and phytanic acid (Gordon et al., 1985; Lowe et al., 1987; Thumser and Wilton, 1996; Wolfrum et al., 1999). In addition, it is associated with the ER membrane of enterocytes where it plays a role as a budding initiator protein for PCTV, indicating its influence on CM synthesis/secretion (Neeli et al., 2007; Siddiqi et al., 2010). Accordingly, *L-FABP* null mice were protected against diet-induced obesity and hepatic steatosis (Newberry et al., 2006). On the other hand, investigators did not record any overt growth delay or failure to gain weight in chow-fed *L-Fabp*^–/–^ mice (Martin et al., 2003; Newberry et al., 2003). More startling is the finding that female *L-Fabp*^–/–^ mice develop a striking obesity phenotype in administrating a semisynthetic diet supplemented with CHOL (Martin et al., 2006). To resolve these conflicting data, different fat diets are administered to female *L-Fabp*^–/–^ mice (Newberry et al., 2008). In contrast to high-polyunsaturated FA, high-saturated fat dramatically protected against obesity and hepatic steatosis, which is indicative that L-FABP functions as a metabolic sensor depending on the type of FA. Later, it has been demonstrated that *L-FABP*^–/–^ mice are characterized by a modest MG trafficking defect and defective mucosal FA oxidation (Gajda et al., 2013). A recent study has shown that low L-FABP expression compromises initial uptake rate of FA and also reduces basolateral TG secretion (Rodriguez Sawicki et al., 2017).

Noteworthy, *L-FABP* gene polymorphisms are also associated with FA metabolism particularly in the liver (Richieri et al., 1994). For example, a negative association has been detected between *FABP1 T94A* and plasma TG levels, probably as a consequence of negative interference of the *T94A* variant with FA binding in humans (Fisher et al., 2007; Gao et al., 2010). In line with this assumption, *T94A* substitution markedly altered the human *L-FABP* structure and stability, along with conformational and functional response to fibric acid derivatives (fibrates), a medication lowering blood TG levels and reducing the liver VLDL production (Martin et al., 2013). It is important to point out the binding of L-FABP with PPARα, resulting in ligand transfer and PPARα transcription of multiple proteins in FA metabolism in mouse primary hepatocytes, with a net effect of lessening plasma TG (Velkov, 2013). Whether similar actions of *T94A* variant apply to the small intestine is not known despite the abundant L-FABP content in the small intestine.

### SR-BI Alteration and Intestinal Lipid Secretion

A few groups have detected an association of intestinal lipid absorption with SR-BI, a cell-surface glycoprotein expressed in the apical microvillus membrane. *SR-BI* knockdown results in decreased FA influx and CM export in Caco-2 cells (Levy et al., 2004), whereas *SR-BI* overexpression has led to enhanced dietary CHOL absorption in mice (Bietrix et al., 2006). Other studies have reported raised apical CHOL uptake by Caco-2 cells using SR-BI-blocking antibody and by small inhibitory RNA (Cai et al., 2004). However, these negative findings should be considered with caution since the extracellular loops of SR-BI are efficient receptors for intestinal mixed micelles, and the properties and composition of micellar solution represent a key factor governing micelle interactions with intestinal SR-BI (Goncalves et al., 2015). Precautions do apply since recent works underline that intestinal SR-BI is a critical regulator of CM transport (Lino et al., 2015) and liposoluble vitamins (Mardones et al., 2002; Reboul et al., 2005; van Bennekum et al., 2005). In fact, acute administration of BLT-1, an SR-BI inhibitor, to hamsters and rats significantly lowers postprandial plasma TGs and CM without effect on CHOL accumulation (Lino et al., 2015). Therefore, the authors suggest that intestinal SR-BI is more involved in postprandial TG handling than in CHOL uptake.

### NPC1L1 Alteration and Intestinal Lipid Secretion

As mentioned previously, NPC1L1 mediates CHOL trafficking from the apical microvillus membrane to the ER (Sane et al., 2006; Field et al., 2007; Nakano et al., 2019). Since free and esterified CHOL constitute moieties of CM components, and since NPC1L1 gene expression in the gut is closely correlated with CM-CHOL (Lally et al., 2007), it is believed that NPC1L1-mediated CHOL supply to CM formation plays a role in lipid assimilation process in enterocytes. In line with this assumption, the specific inhibitor of NPC1L1 is able to reduce postprandial Apo B-48 output in hamsters and lessen CM secretion from the intestine of mice fed a Western diet (Sandoval et al., 2010). Interestingly, NPC1L1 impedes CHOL esterification and intracellular CM vesicle trafficking in enterocytes in response to ezetimibe, which suggests that NPC1L1 participates in a control mechanism for competent CM packaging and output by restraining intracellular CHOL movement at a cellular level (Nakano et al., 2020).

### ABCG5/G8 Alteration and Intestinal Lipid Secretion

Little information is available on the role of ABCG5/G8 in intestinal lipid transport and CM production. However, it is important to note that ABG5/G8 deficiency causes hypertriglyceridemia by increasing intestinal absorption, stimulating hepatic TG production, and lowering plasma TG catabolism in mice with ABCG5/G8 deficiency (Mendez-Gonzalez et al., 2011). In this study, intestinal absorption and secretion of TG were enhanced in ABCG5/G8 null mice, but TG secretion appeared to be greater than TG absorption.

### ACAT2 Alteration and Intestinal Lipid Secretion

Elegant studies demonstrated that ACAT2, a CHOL-esterifying enzyme residing in the ER membrane, increases CHOL absorption efficiency by providing CE for CM packaging and exocytosis into lymph (Nguyen et al., 2012). However, very poor data are available to draw conclusion on the role of ACAT2 in CM assembly and secretion.

### DGAT1 Gene Defects and Intestinal Symptoms

As mentioned before, the *DGAT1* gene encodes DGAT1 protein, a microsomal enzyme with an abundant expression particularly in the small intestine (Cases et al., 1998; Haas et al., 2012). In humans, DGAT1 catalyzes the final step in TG synthesis using DG and FA-CoA, supporting lipid absorption (Yen et al., 2008). Given the lack of *DGAT2* expression in the human intestine (Haas et al., 2012), mutations in *DGAT1* gene may cause conceptually various disorders (Table 1). In fact, patients with molecular aberrations in *DGAT1* exhibited protein-losing enteropathy, a congenital diarrheal disorder with failure to thrive in early infancy (Haas et al., 2012; Stephen et al., 2016; Gluchowski et al., 2017; Ratchford et al., 2018; van Rijn et al., 2018; Ye et al., 2019; Xu et al., 2020). Loss-of-function as a consequence of *DGAT1* variations may also cause elevated fecal alpha-1-antitrypsin, high TGs, vomiting, low albumin, elevated transaminases, and low IgG (Haas et al., 2012; Stephen et al., 2016; Gluchowski et al., 2017; Schlegel et al., 2018). Although lipotoxicity in the intestinal epithelium leading to mucosal injury may explain clinical features in response to *DGAT1*mutations, further studies are required to explore the mechanisms. Fat-restricted diet constitutes an appropriate nutrition therapy.

### DGAT1 vs. DGAT2 in Intestinal Lipid Output and Metabolism

Although *DGAT1* and *DGAT2* genes in mice are expressed in enterocytes (Buhman et al., 2002; Uchida et al., 2013), there was no evidence of overt fat malabsorption in *DGAT1* null mice (Buhman et al., 2002). DGAT1 was not essential for TG absorption and CM synthesis even if a high-fat diet was administered in mice. In fact, *DGAT2* may compensate for *DGAT1* deficiency. Paradoxically, *DGAT1* KO mice displayed resistance to the obesogenic effects of a high-fat diet (Buhman et al., 2002)) and the selective inhibitor JTT-553 of *DGAT1* was able to eliminate the rise of plasma TG and CM in rats after olive oil loading (Tomimoto et al., 2015). By contrast, *DGAT2*^–^*^/^*^–^ mice die within a few hours, likely due to extremely low whole-body TG content and an impaired skin barrier, suggesting a divergent function for the two enzymes (Stone et al., 2004). To determine the specific contribution of each of them on the intestine phenotype, *DGAT1* was only expressed in the gut, and its overexpression did not alter TG secretion compared to wild-type mice (Lee et al., 2010). On the other hand, mice with intestine-specific overexpression of *DGAT2* have higher intestinal TG (Uchida et al., 2013). Another study was able to demonstrate that *DGAT1* and *DGAT2* function coordinately to regulate the process of dietary fat absorption by preferentially synthesizing TG for incorporation into distinct subcellular TG pools in enterocytes (Hung et al., 2017). Definitely, significant divergences characterize the human and mouse species, especially taking into account that DGAT1 is central in human intestinal and DGAT2 is the major enzyme of TG synthesis in mice.

As *DGAT2* shares no sequence homology with the members of the *DGAT1* family, it is important to examine carefully *DGAT2* polymorphisms as we have done for *DGAT1*. When obese children and adolescents and 94 healthy underweight controls were screened for polymorphisms, 15 DNA variants are detected: 4 coding non-synonymous exchanges (p.Val82Ala, p.Arg297Gln, p.Gly318Ser, and p.Leu385Val) and 10 fully synonymous (c.-9447A > G, c.-584C > G, c.-140C > T, c.-30C > T, IVS2-3C > G, c.812A > G, c.920T > C, IVS7+23C > T, IVS7+73C > T, and ^∗^22C > T) (Friedel et al., 2007). The authors do not find (i) an association between variants or haplotypes and the genomic region of *DGAT2*, and (ii) an important role of common genetic variation in *DGAT2* for the development of obesity. On the other hand, using whole-exome sequencing and biological function examination, an obese subject carried one loss-of-function mutation in FA amide hydrolase and one loss-of-function mutation in *DGAT2* (Ning et al., 2017). While inactivation of the former promotes obesity, *DGAT2* modification reduced body weight. This is an interesting investigation documenting an interaction model of genetic variants in two distinct genes in relation with obesity modulation. Finally, mutation of *DGAT2* leads to Charcot-Marie-Tooth disease, an autosomal-dominant axonal neuropathy with low serum TG concentrations (Hong et al., 2016). Accordingly, mutant DGAT2 overexpression of the mutant DGAT2 overexpression significantly inhibited the proliferation of mouse motor neuron cells (Table 1).

### MGAT Alteration and Intestinal Lipid Secretion

While *MGAT1* expression is absent in the small intestine (Cases et al., 1998, 2001; Yen et al., 2002), *MGAT2* expression is abundant in mouse gut (Yen et al., 2002) and its activity correlates with the rate of MG absorption (Yen et al., 2015). However, the deletion of *MGAT2* did not result in a change in normal quantities of fat absorbed from the small intestine aside from an increased energy expenditure noted in knockout mice (Yen et al., 2009; Nelson et al., 2011). Nevertheless, ablation of *MGAT2* specifically in mouse intestine disturbed intestinal TG metabolism and delayed fat absorption (Nelson et al., 2014). In these experimental conditions, the animals were protected against diet-induced weight gain and associated comorbidities.

### Angiopoietin-Like Protein 4 (ANGPTL4) and ANGPTL3 Alterations and Intestinal Lipid Secretion

ANGPTLs constitute a group of proteins, which share structural similarity with angiopoietins, but the absence of the requisite domains do not allow them to bind with the classical angiopoietin receptors, Tie1 or Tie2 (Li and Teng, 2014) (Table 1). The eight members of ANGPTL family play important metabolic roles in diverse biological and pathological processes, including dyslipidemia, IR and wound healing (Basu and Goldberg, 2020). Both ANGPTL3 and ANGPTL4 inhibit lipoprotein lipase (LPL) activity through unfolding and destabilization, leading to its degradation (Sukonina et al., 2006). ANGPTL3 is a 70 kDa protein mostly expressed and secreted by the liver and to a lesser extent by the kidney (Conklin et al., 1999; Romeo et al., 2009), whereas ANGPTL4 glycoprotein is a smaller protein (∼45–65 KDa), which is produced in many cells and tissues, including adipose tissue, liver, intestine, and muscle (Yoon et al., 2000). Although both proteins inhibit LPL activity and raise plasma TG levels, they are regulated by physiological states and different nuclear receptors (Ge et al., 2005). In human, homozygous loss of *ANGPTL3* function leads to familial combined hypolipidemia characterized by low plasma levels of TGs, HDL-CHOL, and LDL-CHOL (Shimamura et al., 2007; Xu et al., 2018). Silencing of *ANGPTL3* in mouse models and in human hepatoma cells result in reduced output and increased uptake of Apo B-containing lipoproteins (Xu et al., 2018), thereby contributing to low LDL-CHOL observed in mice and humans with genetic *ANGPTL3* deficiency (Musunuru et al., 2010). Similarly, *ANGPTL4 null* mice have decreased plasma TG concentrations, whereas mice overexpressing *ANGPTL4* have raised plasma TG levels (Mandard et al., 2006; Lichtenstein et al., 2007). Interestingly, the ablation of *ANGPTL4* caused perturbations of intestinal lymphatics, which worsened after feeding a high-fat diet (Desai et al., 2007). Moreover, *ANGPTL4* was capable of impeding dietary fat digestion via inhibition of pancreatic lipase whereas its deletion increased fat mass LPL, especially with a ANGPTL4 gene variant for a loss-of-function that leads to hypolipidemia with a reduction of TG-containing lipoproteins (VLDL) and CHOL-carrying lipoproteins such as LDL and HDL (Koishi et al., 2002; Shimizugawa et al., 2002; Teslovich et al., 2010). The complete *ANGPTL3* deficiency was associated with a highly reduced postprandial hypertriglyceridemia, probably due to an accelerated catabolism of intestinal derived CM secondary to the increased LPL activity (Minicocci et al., 2016).

## Conclusion

Several advances have been made in our understanding of factors responsible for congenital fat malabsorption syndromes. The information currently at hand has led to map out the route of intestinal lipid transport. We certainly appreciate more the mechanisms controlling intra-enterocyte lipid trafficking and CM formation, but there is still a paucity of knowledge related to the processes essential for its extrusion from the Golgi apparatus and absorptive cells. Probably, the delineation of additional genetic defects in the future will lead to a full characterization of the sequential events crucial for CM packaging and output.

## Author Contributions

EL and SS wrote and edited the manuscript. JFB have made substantial contributions to the manuscript. All authors approved the final version of the manuscript.

## Conflict of Interest

The authors declare that the research was conducted in the absence of any commercial or financial relationships that could be construed as a potential conflict of interest.

## Abbreviations

ABCG5: ATP-binding cassette G5
ABL: Abetalipoproteinemia
ACAT2: Acylcholesterol acyltransferase-2
ANGPTL4: Angiopoietin-like protein 4
Apo: Apolipoprotein
CE: Cholesteryl ester
CHOL: Cholesterol
CM: Chylomicron
CRD: Chylomicron retention disease
DGAT: Diacylglycerol transferase
EFA: Essential fatty acid
ER: Endoplasmic reticulum
FA: Fatty acid
FATP4: Fatty acid transport protein 4
FHBL: Hypobetalipoproteinemia
GI: Gastrointestinal
GTPase: Guanosine triphosphatase
HDL: High density lipoprotein
I-FABP: Intestinal fatty acid-binding protein
IR: Insulin resistance
KO: Knockout
LDL: Low density lipoprotein
LPL: Lipoprotein lipase
MG: Monoglyceride
MGAT: Monoacylglycerol transferase
MTTP: Microsomal triglyceride transfer protein
NPC1L1: Niemann-Pick C1-Like 1
PCTV: Pre-CM transport vesicle
PL: Phospholipid
SR-BI: Scavenger receptor-BI
TG: Triglyceride
VLDL: Very-low density lipoprotein.
